# Commotio Cordis in 2023

**DOI:** 10.1007/s40279-023-01873-6

**Published:** 2023-06-29

**Authors:** Theodore Peng, Laura Trollinger Derry, Vidhushei Yogeswaran, Nora F. Goldschlager

**Affiliations:** 1 Division of Hospital Medicine, Department of Medicine, University of California San Francisco, San Francisco, CA USA; 2 Division of Cardiology, Department of Medicine, University of Washington, Seattle, WA USA; 3 Division of Cardiology, Department of Medicine, University of California San Francisco, San Francisco, CA USA; 4 Division of Cardiology, Department of Medicine, Zuckerberg San Francisco General Hospital, San Francisco, CA USA

## Abstract

Since the nationally televised cardiac arrest of American National Football League player Damar Hamlin in January 2023, commotio cordis has come to the forefront of public attention. Commotio cordis is defined as sudden cardiac arrest due to direct trauma to the precordium resulting in ventricular fibrillation or ventricular tachycardia. While the precise incidence of commotio cordis is not known due to a lack of standardized, mandated reporting, it is the third most common cause of sudden cardiac death in young athletes, with more than 75% of cases occurring during organized and recreational sporting events. Given that survival is closely tied to how quickly victims receive cardiopulmonary resuscitation and defibrillation, it is crucial to raise awareness of commotio cordis so that athletic trainers, coaches, team physicians, and emergency medical personnel can rapidly diagnose and treat this often-fatal condition. Broader distribution of automated external defibrillators in sporting facilities as well as increased presence of medical personnel during sporting events would also likely lead to higher survival rates.

## Key Points

**Table:** 

Commotio cordis is defined as sudden cardiac arrest due to blunt trauma to the left chest area directly over the heart, and rapid recognition and intervention is crucial to improve chances of survival.
This review identified a need for improved access to existing commotio cordis and sudden cardiac death registries, along with more standardized processes for reporting new cases of commotio cordis by athletic trainers, coaches, team physicians, and other healthcare professionals.
Increased distribution and improved maintenance of automated external defibrillators (AEDs) in sporting facilities as well as dedicated training in the use of AEDs would likely lead to higher survival rates for commotio cordis as well as other causes of sudden cardiac death.

## Introduction

On Monday, 2 January 2023, Damar Hamlin of the Buffalo Bills collapsed just 9 min into an American National Football League (NFL) game against the Cincinnati Bengals. Worldwide, millions of spectators witnessed Hamlin take a direct hit to the chest, after which he stood up, took a few steps, then promptly collapsed on the field. First responders quickly responded to his collapse as a cardiac arrest. Initially, there was speculation about the etiology of Hamlin’s cardiac arrest, with theories ranging from a vaccine-related effect to a trauma-related cardiac arrest. Given that all NFL players receive a mandatory in-depth medical evaluation, including an electrocardiogram (ECG), prior to being allowed to compete, a leading theory was that Damar Hamlin collapsed due to commotio cordis (CC). CC is the third most common cause of sudden cardiac death (SCD) in young athletes after hypertrophic cardiomyopathy and congenital coronary artery anomalies (such as anomalous left main coronary artery from right anterior sinus of Valsalva) [1–3]. Because 21 million viewers watched this nationally televised NFL football game, CC has come to the forefront of public attention, with many wondering how this could happen to professional athletes who are perceived to be among the most physically conditioned and health-conscious individuals [2, 4].

CC occurs most commonly in competitive rather than recreational sports, and is defined as a mechanical, blunt trauma to the anterior chest wall that can impact the heart and result in abnormal electrical conduction that leads to a cardiac arrest. Fewer than 20 cases are reported annually in the USA [5]. Initial survival rates reported from the 1970s to 1990s were low, ranging from 10% to 15%. However, with improved cardiopulmonary resuscitation (CPR) efforts and awareness, survival rates in registry data have been reported to have increased to 58% over the past decade [6]. In this review article, we highlight this important cause of SCD in athletes and provide athletic trainers, coaches, team physicians, and other medical personnel with updated information on the epidemiology, pathophysiology, diagnosis, and management of CC.

## Epidemiology

Commotio cordis, which is translated from Latin as “agitation of the heart,” was described as early as 1707 in the book “De Subitaneis Mortibus” by the papal physician Giovanni Maria Lancisi [7, 8]. One of the earliest presumed cases of CC comes from a French case report published in 1876 in which a man fell directly on his chest in a workplace accident and died immediately. Autopsy demonstrated no cardiac structural damage other than bruising of the anterior chest wall [9]. In 1898, a British newspaper reported another one of the first presumed cases of CC in a child in which a 13-year-old boy died after being struck in the chest by a cricket ball [10]. While the majority of reported cases of presumed CC in the early 1900s are found in German journals and involve workplace accidents, by the 1970s there was an increase in reports of SCD in young children and adolescents participating in sports [9].

In 1995, the National Commotio Cordis Registry (NCCR) was established in the USA to gather prospective and retrospective information about cases of CC to increase understanding of the condition and improve prevention efforts (Table 1) [11]. Over the last three decades, there has been a significant increase in public awareness and interest in CC, which may be attributed to the increase in nationally televised sporting events and the rise of social media [5, 12].

**Table 1:** Commotio cordis: relevant registries, injury reporting systems, and databases

Registry	Website	Definition of registry	Number of patients	Comment
US National Commotio Cordis Registry (NCCR) [59]	Not publicly available	National commotio cordis registry based at the Minneapolis Heart Institute Foundation	216 (as of 2012)	Registry attempts to include all US cases of commotio cordis regardless of age or participation in sports. Unclear method for new submissions; no specific webpage on Minneapolis Heart Institute Foundation website
US National Registry of Sudden Death in Athletes [19]	Not publicly available	National sudden death registry of young athletes aged 19 ± 6 years	2406 (as of 2011)	Registry includes deaths of athletes “engaging in an organized team or individual sport requiring regular training and competition.” Deaths “occurring in club or intramural sports or resulting from automobile accidents, cancer, and other systemic diseases” are not included
National Center for Catastrophic Sport Injury Research (NCCSIR) [60]	http://nccsir.unc.edu/about/	National registry of all catastrophic injuries and illnesses related to participation in organized sports based at the University of North Carolina at Chapel Hill	2958 (as of 2021)	Releases annual reports on sports-related catastrophic injuries and illnesses at the collegiate, high school, and youth levels. The NCAA reports cases directly through the NCCSIR
Fédération Internationale de Football Association Sudden Death Registry (FIFA-SDR) [61]	https://www.uni-saarland.de/fakultaet-hw/fifa/en/registry.html	International sudden death and sudden cardiac arrest registry of soccer players based at the Institute of Sports and Preventive Medicine in Saarbrucken, Germany	617 (as of 2018)	Registry includes athletes participating in “football-specific exercise (warm up, training, match) or within 1 h after cessation of activity”
Cardiac Arrest Registry to Enhance Survival (C.A.R.E.S) [62]	https://mycares.net	National cardiac arrest registry based at Emory University	N/A	Registry specifically excludes cardiac arrest cases due to traumatic injury
Cardiac Risk in the Young Center for Cardiac Pathology [71]	https://www.c-r-y.org.uk	National database of sudden cardiac death based in St. George’s University of London	7675 (as of 2022)	Registry includes deaths from the UK

Although some organizations, including the NFL, mandate the reporting of injuries, there is currently no standardized, mandatory reporting of SCD in athletes [13]. This lack of standardized reporting makes it challenging to evaluate the exact incidence of CC [14–16]. As of July 2012, there were 216 cases of CC in the NCCR, with all cases meeting the following inclusion criteria: (1) a witnessed blunt, nonpenetrating blow to the precordium followed immediately by cardiovascular collapse, (2) detailed documentation of events, (3) no evidence of structural damage to the heart on autopsy in nonsurvivors, and (4) absence of underlying cardiovascular abnormalities [6]. Approximately 10–20 cases have historically been reported to the NCCR each year [11, 17]. Over the last few years, there has been a decline in the number of reported cases to the NCCR, which may be due to a decrease in the incidence of these events and/or a reduction in the number of sporting events in the setting of the COVID-19 pandemic [18]. However, the number of new cases of CC reported to the NCCR since July 2012 is not publicly available.

Despite being the third most common cause of SCD in young athletes under 35 years of age, CC accounted for 7% of cases in the US National Registry of Sudden Death in Athletes as of 2011 (Table 1) [2, 3, 19]. Based upon the NCCR, 95% occur in males, with an average age of 15 ± 9 years, ranging from 0.2 (2 months) to 51 years of age [6]. A recent 2023 review of the Cardiac Risk in the Young (CRY) center for cardiac pathology’s database of more than 7000 cases of SCD found that commotio cordis was significantly more common in adolescent athletes (5%) than nonathletes (1%) [20]. It is theorized that younger children and adolescents are at higher risk for CC due to their thinner, more pliable chest walls, which are less able to withstand a direct blow [21]. This may explain why there are fewer cases of CC in older athletes with more developed chest walls.

**Fig. 1:**
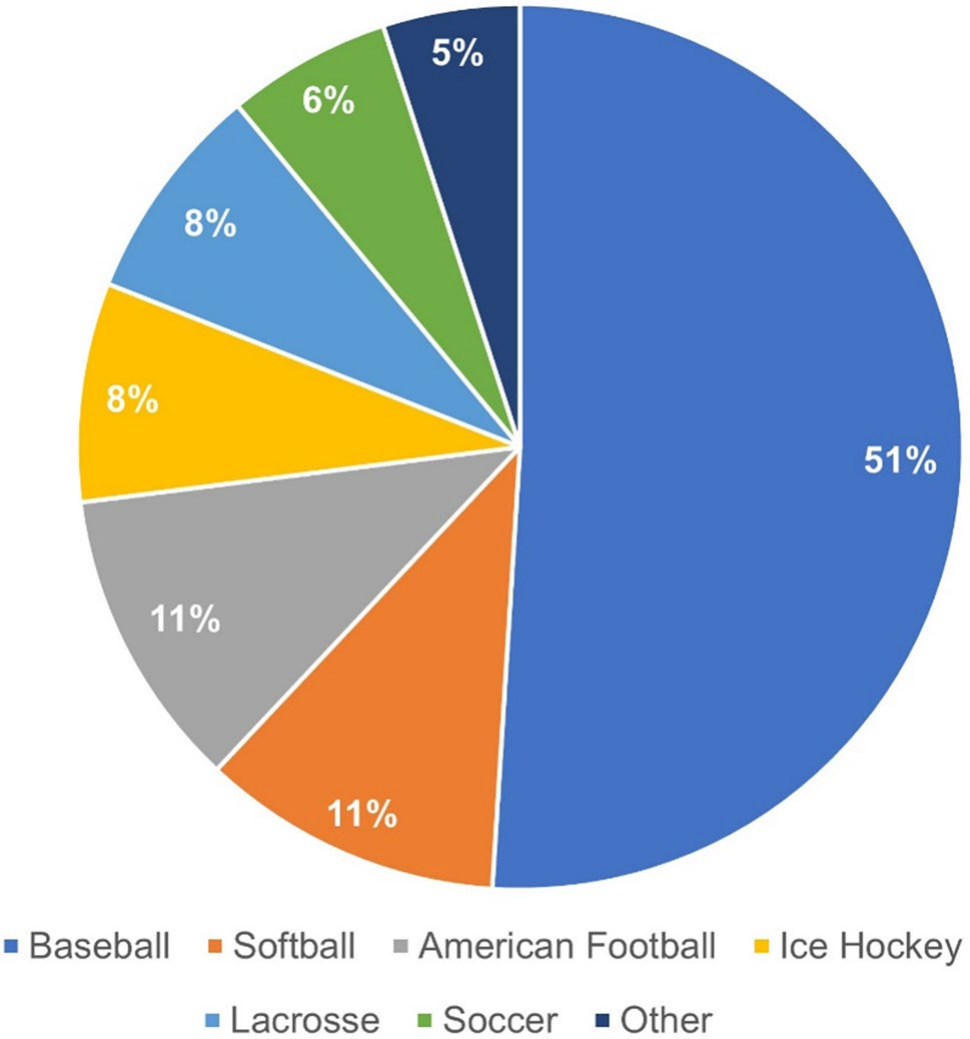
Most common sports in which commotio cordis has been reported as of 2013. The percentages shown reflect reported cases described in references [6, 11, 12, 16, 23, 24]

More than 75% of cases occurred during sports, with two-thirds of those cases occurring in organized competitive sports (e.g., baseball, American Football, soccer) and the remaining one-third in recreational sports (e.g., sports played at home, family gatherings, or the playground) [6, 11, 22]. Baseball (51%) remains the most common sport for CC, followed by softball (11%) and American Football; a breakdown of the most common sports in which CC events occur is shown in Fig. 1. The vast majority of cases resulted from a blow to the chest from a projectile, such as a ball or puck, although cases also occurred after direct physical contact or collisions between competitors [6, 11]. The remaining 25% of cases reported in the NCCR were unrelated to sports and ranged from violent attacks, fights, and traffic accidents to atypical events, such as when a young child was struck in the chest by the head of her dog [11, 12].

In a 2009 retrospective review of deaths in 1866 young athletes over a 27 year period recorded in the US National Registry of Sudden Death in Athletes, 30 out of 65 CC deaths were reported to have occurred during baseball. In this registry, CC was diagnosed based on autopsy findings and history (e.g., the circumstances of collapse, which were often derived from written or verbal accounts through interviews with family members, coaches, and other witnesses) [2]. In the USA, there were a total of seven reported cases of CC during American Football games between 1990 and 2010. In these seven cases, four occurred during a game and three occurred during practice. All cases of CC resulted from direct impact to the chest while tackling or receiving a tackle during organized sporting events [25].

The Fédération Internationale de Football Association (FIFA) Sudden Death Registry recorded 617 cases of SCD in soccer players across 67 countries between 2014 and 2018 (Table 1) [26]. Of these, a cause of death was documented in 211 cases. CC was confirmed in seven cases by autopsy and highly suspected in seven others based on clinical history (e.g., direct impact to the precordium resulting in cardiac arrest), contributing to 7% (14/211) of total cases in this registry. In 11 of these 14 cases, the soccer ball hit the chest, and in three there was a tackle with impact to the precordium. Overall, there was a 43% survival rate of CC cases in this registry [25].

## Pathophysiology

Commotio cordis leads to a lethal arrhythmia that results from nonpenetrating, blunt trauma over the precordium, or anterior chest wall [12]. Based on animal models and data from initial ECGs recorded in the field or in the emergency department (ED), ventricular fibrillation (VF) and ventricular tachycardia (VT) are the most common arrhythmias [11, 12]. Of note, there are two case reports of atrial fibrillation resulting from blunt trauma to the chest wall in athletes; however, the ECG was obtained in the ED 25 min after the collision in one case and in the field in the second [27, 28]. Therefore, it is unclear if these specific cases are true episodes of CC given that VF/VT were not recorded immediately at the time of the event.

Experimental laboratory models conducted using swine, dogs, and rabbits have helped to elucidate four specific factors that influence whether blunt trauma will lead to CC: (1) location, (2) timing, (3) projectile structure, and (4) velocity [29]. Location of the trauma must be directly over the anatomic position of the heart [12, 23, 29]. Precordial bruising is frequently visible on CC patients [23, 30]. There are no reported cases of CC in humans or experimental models of trauma outside of the precordium [29, 31].

**Fig. 2:**
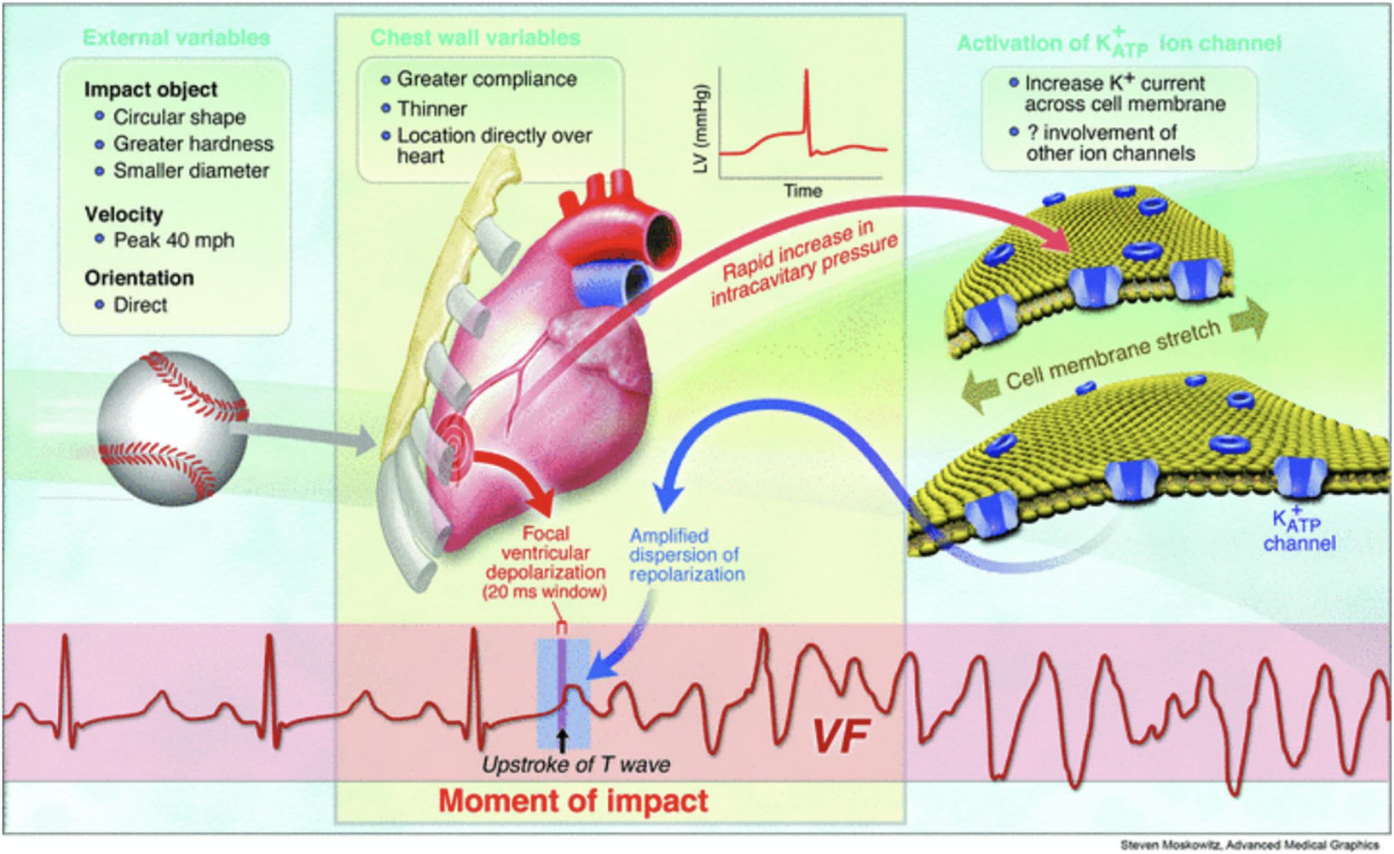
The variables associated with commotio cordis (CC) together with the proposed mechanism for CC (Reproduced from Link and Estes [37], with permission)

The next important factor is the timing of the trauma in relation to the cardiac cycle. In the swine model, impacts occurring only during a narrow (15 ms) window of ventricular repolarization (the upstroke of the T wave, which accounts for only 1% of the cardiac cycle duration) resulted in VF [32]. A six-lead electrocardiogram showing the electrocardiographic change from sinus rhythm to VF following impact in the swine model is seen in Figure 2 of reference 17 (not published here due to copyright) [17]. During this narrow interval of the swine cardiac cycle, there was increased heterogeneity of repolarization across the myocardium, and any incidence of premature ventricular depolarization in this brief time period could lead directly to VF [31]. It is hypothesized that this premature ventricular depolarization resulted from the activation of stretch-sensitive K+ ATP channels in the myocardium following blunt trauma. These same swine models demonstrated that left ventricular intracavitary pressure rose to between 250 and 450 mmHg (mean 128 mmHg on echocardiography, depending on size of swine) following a precordial strike, likely activating these stretch-sensitive ion channels and predisposing cardiac tissue towards arrhythmogenic abnormalities [31–33]. In swine, when trauma occurred outside of this brief window, other arrhythmias occasionally resulted, such as transient complete heart block and left bundle-branch block [32]. Interestingly, several cases of heart block in humans have been reported following direct precordial trauma [34, 35]. It is possible that the aforementioned cases of documented atrial fibrillation in humans may have resulted from trauma occurring outside the 15 ms interval.

The structure and velocity of the projectile also influence the likelihood of CC. While there is a wide range of objects that have been reported to be responsible for cases of CC in retrospective studies of humans (e.g., sled, boxing glove, plastic bat), harder, smaller, sphere-shaped projectiles have been found to be the most likely to induce lethal VF for reasons that have not been elucidated [32, 36]. Experimental models in swine also demonstrated that projectiles traveling at 40 miles per hour were most likely to induce VF, whereas velocities above 50 miles per hour were more likely to cause structural damage and cardiac contusion rather than arrhythmias [21]. The variables that are associated with CC are shown in Fig. 2.

## Presentation

While commotio cordis most commonly occurs in organized sports, registry data show that it can occur with any blunt-force trauma directly over the heart, such as when an infant was struck in the chest by the head of her pet dog or a when a young boy “instantly died” after being struck in the chest by a circular sledding saucer [12]. In a review of 25 cases, 12 patients collapsed immediately on direct impact while 13 briefly remained conscious and active before cardiac arrest. Of the 13 patients who did not immediately collapse with direct impact, eye opening, attempting to or rising to standing, verbalizing, and/or making an attempt to continue play (e.g., throwing a ball, walking, skating) were observed. It is important to note that 20% of CC victims are still able to stand and walk for several seconds after the blow, which may be due to their ability to tolerate VF for a short time period [11]. It is also important to recognize that there are no prodromal symptoms [23].

## Diagnostic Evaluation

Diagnosis of CC is made when the following are present: (1) a witnessed event of direct chest wall contact occurred and was followed by near-immediate cardiac arrest, (2) ECG recordings at or around the cardiac arrest demonstrated VF/VT, and (3) subsequent studies confirmed the absence of underlying structural heart disease, ischemia, conduction system disease, or myocardial trauma in postarrest diagnostic studies on survivors or autopsy [9, 11]. Other causes of SCD, including coronary artery disease, cardiomyopathy, congenital heart disease, valvular disease, genetic abnormalities, and medications or other substances, should be excluded in the diagnostic evaluation and/or autopsy.

Diagnostic evaluation should include a detailed history, physical examination, and diagnostic studies. The history should include a detailed understanding of whether there were prodromal symptoms and history obtained from witnesses; past medical history; family history of cardiovascular disease, including sudden death; social history, including consumption and/or recreational use of other substances (e.g., alcohol, illicit drugs, over-the-counter medications, performance-enhancing drugs, herbs); and medications. Physical examination should include a cardiopulmonary assessment to exclude structural and myocardial disease. Diagnostic studies should include basic laboratory studies (e.g., complete blood count, chemistry), toxicology screen, cardiac biomarkers (e.g., troponin, creatinine kinase-MB, B-type natriuretic peptide), ECG, chest imaging (e.g., chest X-ray, chest computed tomography), and cardiac imaging (e.g., echocardiogram, cardiac magnetic resonance imaging). A cardiac catheterization may be recommended. ECG data and imaging studies should be reviewed to identify the presence of channelopathies (e.g., Brugada syndrome, long-QT syndrome) and structural heart disease, respectively. In a review of rhythms documented in the NCCR up to September 2001, postarrest ECG data recorded in the ED were analyzed in 82 of the 128 patients: 33 patients had VF, 3 VT, 2 idioventricular rhythm, 1 complete heart block, and 40 asystole. These recorded ECGs were likely not the initial rhythms at time of cardiac arrest or inciting cause of cardiac arrest [12]. Autopsy should be performed to confirm a diagnosis in SCD nonsurvivors, especially when criminal intent is suspected [5].

## Management

The management of CC should follow guideline-directed management of cardiac arrest [38]. After a cardiac arrest is recognized, the emergency response system should be activated with early automated external defibrillator (AED) use and prompt initiation of high-quality CPR. Since CC cases result in VF/VT, concurrent AED use and CPR are critical for survival [39]. In the swine model of CC, all episodes of VF were successfully terminated with 94% of cases terminated with a single 200 J defibrillation. In these swine models, it was observed that AED shocks applied at 1 min intervals from the traumatic event had a 100% survival rate, compared with only 46% in those applied at 4 min, emphasizing the importance of prompt AED shocks [40].

Initial studies on CC reported survival rates of 10–15%, with data from the NCCR reporting a survival rate of 15% in 2002 [12, 41]. In the updated NCCR registry in 2013, survival had increased almost fourfold to 58%. The high survival rates in the updated NCCR reflected prompt resuscitation times and the situation in which the cardiac arrest occurred. In individuals with a less than 3 min interval between collapse and resuscitation initiation, there was a 40% survival, compared with only 5% in individuals with a delay in resuscitation greater than 3 min [6]. These survival trends and practice improvements also correlate with efforts to improve access to AEDs and improve first responder rates in the general population [42–44]. The American Heart Association recommends that AEDs should be accessible within 5 min of collapse at organized sporting events [45].

It was also observed that participation in competitive sports (in comparison with recreational activity or normal daily activities) has also been associated with survival, likely due to the presence of sports trainers and emergency personnel at the time of the cardiac arrest and shorter resuscitation times [6, 41]. Today, many major league sports teams require that medical personnel be onsite during games. In the NFL, around 30 medical personnel, including certified athletic trainer spotters, orthopedic physicians, and medical (e.g., family medicine, internal medicine, emergency medicine) physicians are required [46–48]. The American National Hockey League (NHL) requires that at least three physicians, two ambulances, and two AEDs be present at all arenas, and American Major League Baseball (MLB) mandates that there be healthcare providers onsite at each of the 28 stadiums [49, 50].

After achieving return of spontaneous circulation (ROSC) in the emergency response algorithm, or while CPR is in process, patients should immediately be transferred to the nearest ED for appropriate management. CC recurrence is extremely rare, with only one reported case of suspected CC recurrence in a young teenage boy with two distinct syncopal episodes following blunt trauma to his chest. In the first episode, he was punched in the mid-chest by a friend, and subsequently lost consciousness and stopped breathing for 10–15 s. In the second episode a year later, he lost consciousness and reportedly turned blue after being struck in the chest by a tennis ball filled with coins (regaining consciousness just as CPR was initiated). Of note, there was no documented evidence of VF in either episode, so whether these cases were truly CC remains unclear [22]. As there are no other data that suggest that survivors of CC are at increased risk of subsequent arrhythmias or sudden cardiac death, implantable cardioverter defibrillators have not been recommended [11, 51]. In individuals who have a CC event in a sport, returning to play is made on a case-by-case basis [41, 52].

In addition to improved resuscitation efforts, increased awareness, early AED application, and prevention and mitigation strategies are being studied [50]. Many sports teams and organized sports leagues use chest barriers as a protective mechanism for CC and other life-threatening sports injuries, and in January 2017, the National Operating Committee on Standards for Athletic Equipment published the world’s first performance standard for chest protection in baseball and lacrosse [53]. However, chest wall protectors have not demonstrated protection against provoking life-threatening arrhythmias in both retrospective CC registry studies and swine models [54, 55]. Safety baseballs, a category of balls used commonly in T-ball, have been demonstrated to reduce the risk of SCD in swine models [21]. Safety balls include tennis balls, rubber balls, cloth balls, and reduced-impact balls [56]. Although these have not been studied to have the same impact in humans, age-appropriate safety baseballs are currently recommended for children up to 13 years old [51].

## Conclusion and Future Directions

### Update on Damar Hamlin

On 11 January 2023, 9 days after his cardiac arrest, Damar Hamlin was discharged from the hospital. Just weeks after his cardiac arrest, he was seen attending the NFL playoff game between the Buffalo Bills and Cincinnati Bengals. Three months later on 18 April 2023, Damar Hamlin was cleared by the NFL and his physicians to resume full football activities, with Hamlin reporting in an interview that the cause of his collapse was CC [57].  His case is a critical reminder of the importance of quick recognition of CC as well as rapid CPR and early defibrillation. Currently, out-of-hospital cardiac arrest victims receive CPR in only 40% of cases and defibrillation in just 10% of cases, indicating that improving public education about these key interventions is needed [58]. While Damar Hamlin survived CC, many young athletes in the NCCR have not, and potential policy changes and precautionary steps need to be taken to improve survival rates.

### Future Directions for Commotio Cordis

The precise incidence of CC in athletes is unknown, despite the presence of both national and international sport-specific registries that track cardiac arrest, SCD, and sport injuries (Table 1) [19, 58, 59–62]. At present, criteria for submission of new cases into existing registries are not standardized, and some registries are difficult to access. Without record keeping and knowledge of the precise incidence, epidemiology, and clinical presentations of CC, it is difficult to develop new rules and policies that improve athlete safety and prevent CC. As a result, we recommend improved reporting by athletic trainers, coaches, team physicians, emergency medical responders, and other healthcare professionals, along with creating accessible, standardized, and electronic processes to facilitate reporting cases of cardiac arrest and SCD in athletes.

While the presence of medical personnel is mandated in many American national sport leagues such as the NFL, NHL, and MLB, as well as in the American National Collegiate Athletic Association (NCAA), this is not the case in many organized high school sport leagues and other amateur sport leagues [63]. Therefore, the presence of at least one medical professional during organized sporting events can be recommended, and these medical professionals should be up to date on advanced cardiac life support (ACLS) and basic life support (BLS) certification. Additionally, it would be reasonable for all coaches, personnel, and players to be trained on CPR and AED usage. There is renewed interest at the state level in the USA to increase hands-on CPR and AED training in either a health or physical education (PE) class as part of the high school graduation requirement. For example, Assembly Member Brian Maienschein of the Seventy-Sixth District of California proposed this requirement in California Assembly Bill 1473 in February 2023 [64]. 

While AED usage has increased in the past two decades, these devices are not uniformly available in school gyms, on athletic fields, or in sporting arenas where organized sports take place [42–44, 65]. Where they are available, published studies show that AEDs are not always well maintained, medical professionals are often not trained on how to use them, and nearly half of coaches and staff are unable to identify where they are located [66]. Distribution and maintenance of AEDs in sporting facilities, dedicated training in using AEDs, and formalized emergency action plans would likely lead to even higher survival rates [15].

Implementing these recommendations will require personnel and funding, and there are now federal legislative efforts and nationwide campaigns to address this. In March 2023, US Representatives Sheila Cherfilus-McCormick and Bill Posey introduced the *Access to AEDs Act*, a bipartisan bill that would provide elementary and secondary schools with funds to “maintain AEDs, strengthen CPR training, and develop cardiac emergency response plans” [67, 68]. In the same month, the NFL together with other American major pro sport leagues, patient advocacy organizations, and leading medical groups such as the American Heart Association launched a nationwide campaign called *The Smart Heart Sports Coalition*. Additional professional organizations such as the Heart Rhythm Society, the American College of Cardiology, and the American Medical Society for Sports Medicine have since joined. The coalition advocates for state governments to implement policies to prevent fatal outcomes from cardiac arrest among high school students and has committed over $1 million to high schools nationwide for CPR and AED education and equipment [69, 70]. Altogether, these efforts will likely result in increased safety for athletes and reduce mortality from CC. 
